# Tract-wise microstructural analysis informs on current and future disability in early multiple sclerosis

**DOI:** 10.1007/s00415-023-12023-3

**Published:** 2023-10-11

**Authors:** Veronica Ravano, Gian Franco Piredda, Jan Krasensky, Michaela Andelova, Tomas Uher, Barbora Srpova, Eva Kubala Havrdova, Karolina Vodehnalova, Dana Horakova, Petra Nytrova, Jonathan A. Disselhorst, Tom Hilbert, Bénédicte Maréchal, Jean-Philippe Thiran, Tobias Kober, Jonas Richiardi, Manuela Vaneckova

**Affiliations:** 1 Advanced Clinical Imaging Technology, Siemens Healthineers International AG, Lausanne, Switzerland; 2 Department of Radiology, Lausanne University Hospital and University of Lausanne, Lausanne, Switzerland; 3 LTS5, École Polytechnique Fédérale de Lausanne (EPFL), Lausanne, Switzerland; 4 Department of Radiology, First Faculty of Medicine, Charles University and General University Hospital, Prague, Czech Republic; 5 Department of Neurology and Center of Clinical Neuroscience, First Faculty of Medicine, Charles University and General University Hospital, Prague, Czech Republic

**Keywords:** Multiple sclerosis, Magnetic resonance imaging, White matter, Demyelinating diseases, Relaxometry

## Abstract

**Objectives:**

Microstructural characterization of patients with multiple sclerosis (MS) has been shown to correlate better with disability compared to conventional radiological biomarkers. Quantitative MRI provides effective means to characterize microstructural brain tissue changes both in lesions and normal-appearing brain tissue. However, the impact of the location of microstructural alterations in terms of neuronal pathways has not been thoroughly explored so far. Here, we study the extent and the location of tissue changes probed using quantitative MRI along white matter (WM) tracts extracted from a connectivity atlas.

**Methods:**

We quantified voxel-wise T1 tissue alterations compared to normative values in a cohort of 99 MS patients. For each WM tract, we extracted metrics reflecting tissue alterations both in lesions and normal-appearing WM and correlated these with cross-sectional disability and disability evolution after 2 years.

**Results:**

In early MS patients, T1 alterations in normal-appearing WM correlated better with disability evolution compared to cross-sectional disability. Further, the presence of lesions in supratentorial tracts was more strongly associated with cross-sectional disability, while microstructural alterations in infratentorial pathways yielded higher correlations with disability evolution. In progressive patients, all major WM pathways contributed similarly to explaining disability, and correlations with disability evolution were generally poor.

**Conclusions:**

We showed that microstructural changes evaluated in specific WM pathways contribute to explaining future disability in early MS, hence highlighting the potential of tract-wise analyses in monitoring disease progression. Further, the proposed technique allows to estimate WM tract-specific microstructural characteristics in clinically compatible acquisition times, without the need for advanced diffusion imaging.

**Supplementary Information:**

The online version contains supplementary material available at 10.1007/s00415-023-12023-3.

## Introduction

In recent years, the development of quantitative magnetic resonance imaging (qMRI) techniques with clinically feasible acquisition times has enabled the characterization of microstructural changes in brain tissue in clinical research settings. In multiple sclerosis (MS), qMRI was shown to provide valuable information, especially with regard to the iron and myelin content [1, 2], both within lesions and normal-appearing tissue [3]. In this context, T1 relaxation time is an effective imaging biomarker known to be associated with demyelination, iron accumulation and axonal loss [4].

Previous studies showed the clinical value of T1 mapping in MS. In particular, lesions with longer T1 values (so-called black holes) were shown to be better associated with disability compared to total lesion volume (TLV) [5]. Further, longitudinal microstructural changes in black holes was shown to be correlated with disability evolution [6]. Likewise, T1 relaxation values were reported to improve disability prediction when measured in normal-appearing brain tissue [7–9].

Not only the extent of the microstructural damage, but also the location of brain damage is known to play an important role in explaining disability [10]. Microstructural alterations in white matter (WM) tracts are of particular interest as different white matter pathways are known to be responsible for different brain functions, hence enabling lesion-to-symptom mapping [11, 12]. In this context, estimating damage based on structural connectivity, which takes into account the location of a lesion, has been shown to improve the correlation with Expanded Disability Status Scale (EDSS) [13–16].

In addition to their potential clinical relevance, white matter tract-based analyses are required to study pathophysiological mechanisms such as Wallerian degeneration [17], previously shown to play a role in MS [18–20]. This retrograde degeneration suggests that with time, the presence of a demyelinating focal lesion on a white matter tract can induce microstructural changes on more distal portions of the tract. Along these lines, previous work showed that the presence of lesions in the pons was inducing microstructural diffusion changes not only at the lesion site but also along the entire affected tract [16]. Furthermore, evaluating microstructural properties in a selection of white matter tracts using T1 relaxometry, was shown to result in significant correlations with specific functional scores and EDSS [21].

To date, there has been little research into the microstructural properties of specific white matter pathways due to its limited feasibility in a clinical setting. In fact, the delineation of white matter tracts relies on high-quality diffusion imaging data, typically characterized by long acquisition times, and model-based tractography algorithms that often result in limited inter-patient comparability.

In this work, we evaluated the distribution and extent of abnormal T1 relaxation times along white matter tracts extracted from a connectivity atlas. Deviations in T1 values were calculated with respect to a normative atlas considering age and sex. The clinical value of the proposed technique was assessed by correlation with EDSS and disability progression over 2 years in two distinct MS cohorts (early MS and progressive MS).

## Methods

### Participants

The cohort included 92 healthy subjects (age 37.3 ± 10.6 years, 63% female), 47 patients with first symptoms suggestive of MS (age 31.8 ± 8.0 years, 75% female), and 52 patients with progressive MS (including 7 primary and 45 secondary progressive MS, age 49.9 ± 7.2 years, 65% female). Detailed demographics and clinical scores are reported in Table 1.

**Table 1:** Demographics and disease characteristics of healthy individuals and MS cohorts

Parameter	Healthy cohort	Early MS	Progressive MS	W (*p*-value)
*N* (% female)	92 (63%)	47 (75%)	52 (65%)	–
Age (years)	37.3 ± 10.6	32.4 ± 8.0	49.8 ± 7.3	2255 (5.9 × 10^–14^)
Disease duration	–	6.3 ± 5.4 months	19.45 ± 7.8 years	2346 (< 10^–16^)
EDSS_0_	–	1.87 ± 0.77	5.20 ± 0.89	2387 (< 10^–16^)
ΔEDSS	–	0.01 ± 0.54	0.24 ± 0.55	1468 (0.037)
Δ*t* [months]	–	24.7 ± 1.3	25.0 ± 3.7	1488 (0.04)
TLV [mL]	–	6.7 ± 8.7	14.3 ± 15.3	1721 (2 × 10^–4^)
TLC [# of lesions]	–	22.2 ± 23.6	28.1 ± 15.9	1615 (0.003)

The results of the Wilcoxon rank-sum test comparison between MS groups are reported in the last column

*EDSS* Expanded Disease Disability Scale; *ΔEDSS* EDSS change in 2 years; *Δt* time elapsed between clinical evaluations; *TLV* total lesion volume; *TLC* total lesion count

Patients and healthy subjects underwent MR examinations at 3T (MAGNETOM Skyra, Siemens Healthcare, Erlangen, Germany) using a standard 20-channel head coil. A 3D magnetization-prepared 2 rapid acquisition gradient echoes (MP2RAGE) sequence was used for T1 mapping [22, 23] in addition to the standard clinical imaging used for lesion detection, consisting of a 3D magnetization-prepared rapid gradient echo (MP-RAGE) and a 3D fluid-attenuated inversion recovery (FLAIR). Detailed sequence parameters are reported in Table 2.

**Table 2:** Relevant MRI acquisition parameters

Parameter	3D MP-RAGE	3D FLAIR	3D MP2RAGE	
			HC and early MS	Progressive MS
Resolution [mm^3^]	1.0 × 1.0 × 1.0	1.0 × 1.0 × 1.0	1.0 × 1.0 × 1.2	1.0 × 1.0 × 1.0
Field of view [mm^3^]	256 × 256 × 176	256 × 256 × 176	256 × 256 × 212	256 × 256 × 224
Undersampling	GRAPPA × 2	GRAPPA × 3	GRAPPA × 3	CS × 4
TA [min]	5:30	3:17	8:22	4:35
TI_1_/TI_2_ [ms]	900/–	1800/–	700/2500	
TE [ms]	–	397	–	
Flip angles [°]	9	–	4/5	
TR [s]	2.3	5	5	
Bandwidth [Hz/Px]	240	781	240	

*MP-RAGE* magnetization-prepared rapid gradient echo; *FLAIR* fluid-attenuated inversion recovery; *MP2RAGE* magnetization-prepared 2 rapid gradient echo; *TA* acquisition time; *TI* inversion time; *TE* echo time; *TR* repetition time; *CS* compressed sensing

Patients were scored using EDSS [24]. The neurological examinations were conducted shortly after (0.9 ± 1.0 months), and 2 years after (24.2 ± 3.0 months) the MR examination. Clinical examinations were always conducted outside any clinical relapse and at least 30 days after the administration of corticosteroids.

In the early cohort, the majority patients received first-line treatments (43/47), while four did not receive any treatment, and eleven patients received second-line treatments after 2 years. In the progressive cohort, most patients were treated with second-line drugs at the time of the first clinical examination (30/52). The precise breakdown of treatment status within both groups is reported in Supplementary Table 1.

To ensure T1 values were consistent between both undersampling techniques and in addition to the analyses conducted in [23], we compared T1 maps and *z*-score maps in a patient with progressive MS, who was examined with both GRAPPAx3 and CSx4 MP2RAGE.

### Preprocessing

A lesion segmentation research application [25, 26] that performs voxel-wise lesion detection, followed by partial volume estimation was used to automatically segment lesions on FLAIR and MP-RAGE images. A threshold was applied to the resulting lesion concentration maps to obtain binary lesion masks as described previously [25]. For each patient, an affine spatial transformation was computed between the MP-RAGE and the MP2RAGE space and applied to the binary lesion mask using Elastix [27]. Total lesion volume (TLV) and count (TLC) were extracted for each patient.

Automated brain segmentation and skull-stripping were performed on the MP2RAGE contrast using the MorphoBox research application [28].

### T1 normative atlas

The calculation of the T1 abnormalities follows the method of Piredda et al. [29] where it is described in more detail. In short, 20 skull-stripped MP2RAGE datasets were randomly selected from the healthy subjects cohort and the skull-striped uniform (UNI) MP2RAGE images were co-registered to build a study-specific template (SST) [30]. Then, spatial transformations between the skull-stripped UNI images data of all subjects in the healthy cohort and the SST were computed and applied to the associated T1 maps. Finally, normative T1 values were established using a linear voxel-wise model of inter-subject variability in T1 values, while considering age (centered at the average age of the healthy cohort) and sex as covariates:

$${\varvec{E}}\left\{T1\right\}= {\beta }_{0}+{\beta }_{\mathrm{sex}}*\mathrm{sex}+{\beta }_{\mathrm{age}}*\mathrm{age}+{\beta }_{{\mathrm{age}}^{2}}*{\mathrm{age}}^{2},$$

with ${\beta }_{0}$ being the model intercept, and sex a categorical variable equal to 1 if the subject is male or 0 if female as detailed in Piredda et al. [29].

### Atlas-based white matter tract maps

**Fig. 1:**
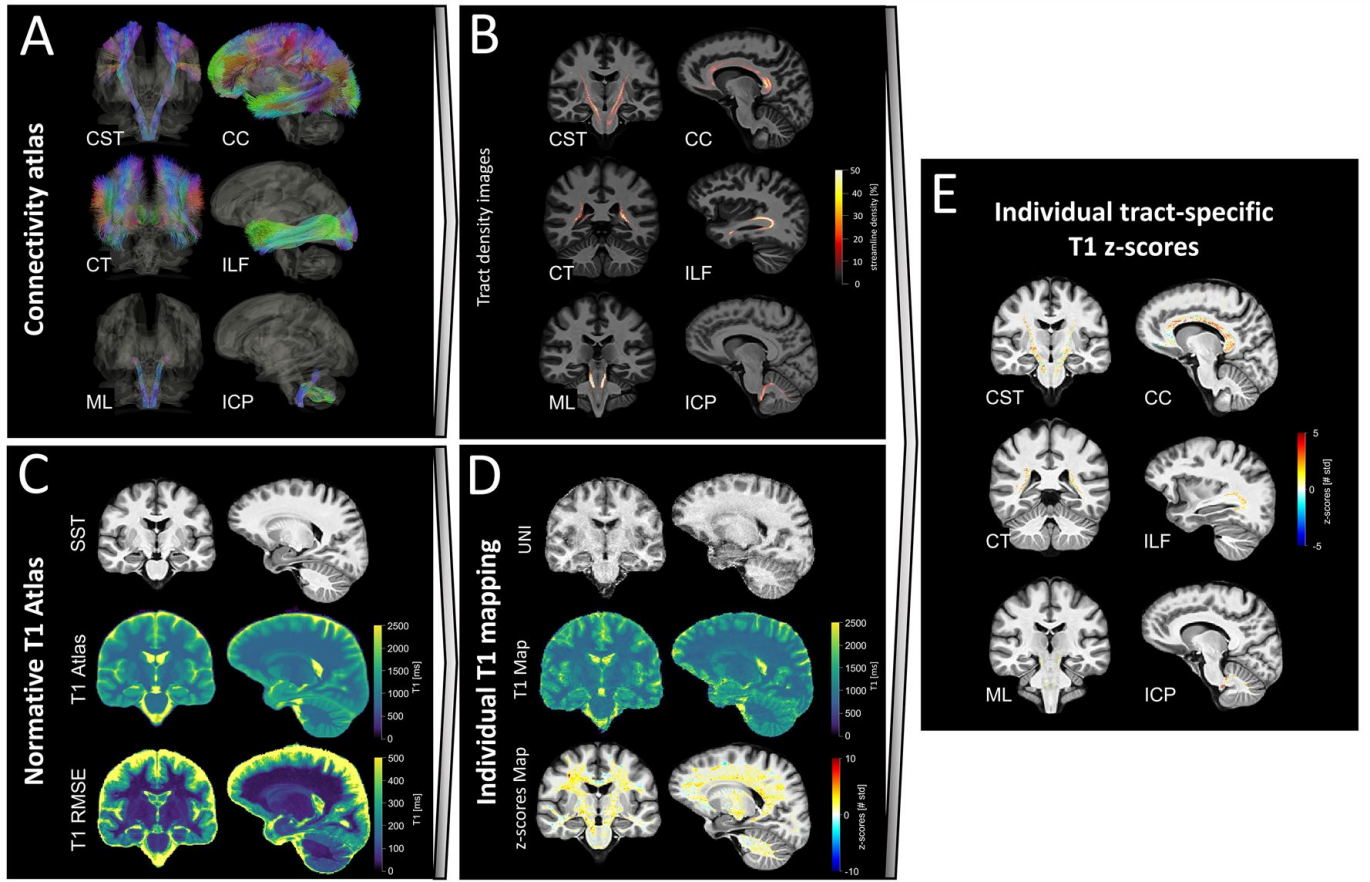
Pipeline used to extract white matter, tract-specific T1 abnormalities shown here for six example white matter tracts. **A** White matter tract streamlines from a connectivity atlas [31]. **B** Normalized tract density images representing the percentage of streamlines passing through each voxel for each individual white matter tract, registered to SST space. **C** T1 normative voxel-wise atlas estimated from the healthy cohort and using sex and age as covariates. **D** Estimation of T1 deviations from the normative atlas in terms of *z*-scores for individual patients.** E** Estimation of tract-specific T1 *z*-scores in each patient. *CST* cortico-spinal tract; *CT* corticostriatal pathway; *ML* medial lemniscus; *CC* corpus callosum; *ILF* inferior longitudinal fasciculus; *ICP* inferior cerebellar peduncle; *SST* spinothalamic tract; *RMSE* root mean squared error; *UNI* uniform MP2RAGE image

Voxel-wise tract density maps, representing the number of streamlines passing through each voxel, were extracted for white matter tracts defined in a tractography atlas [31], using DSI Studio (Fig. 1A, B). Tracts that were defined in both hemispheres were merged into a single tract by summing the density maps voxel-wise, resulting in 37 white matter tracts. A non-linear spatial registration with trilinear B-spline interpolation between the MNI space (where the tractography atlas was originally defined) and the SST was computed using Elastix [27] and applied to all tract density maps. Finally, each map was normalized by its maximum value and binarized using a threshold at 5% to discard voxels with low tract density.

### Extraction of tract-specific *z*-scores

For each patient, the non-linear spatial registration between the skull-stripped UNI image and the SST was computed with Elastix [27] and applied to the associated T1 map. Then, voxel-wise deviations (measured as *z*-scores) from the established T1 normative atlas were computed (Fig. 1C, D), yielding a map which shows how many standard deviations the T1 in each voxel is away from the mean, as described in Piredda et al. [29]. To discard cases with insufficient registration quality, typically caused by the presence of brain atrophy, we computed the mutual information from the joint histogram of the registered anatomical UNI image and the SST. Then, a threshold was set to the fifth percentile of the mutual information estimated on the whole MS cohort, and the so-detected datasets were visually inspected.

White matter tract-specific analysis was performed by superimposing the white matter tract density images and the T1 *z*-score maps of individual patients in the SST space (Fig. 1E). In each tract, we extracted two imaging biomarkers: the average absolute *z*-score value (${\mu }_{|z|}$), representing the magnitude of deviation from the norm, and the number of voxels with an absolute *z*-score larger than 2 (${V}_{\left|z\right|>2}$), representing the spatial extent of the deviation from the norm, both in NAWM and lesions. For comparison, the tract-specific lesion volume (LV) was also computed by estimating the volume of lesions on each tract from the registered lesion mask.

### Statistical analysis

All statistical analyses were performed using RStudio v1.1.456 [32].

Spearman correlations were computed between the tract-specific metrics (LV, ${V}_{\left|z\right|>2}^{\mathrm{NAWM}}$, ${V}_{\left|z\right|>2}^{\mathrm{lesions}}$*,* ${\mu }_{|z|}^{\mathrm{NAWM}},$ and ${\mu }_{|z|}^{\mathrm{lesions}}$) and disability in both MS cohorts and compared to radiological metrics reflecting lesion load, namely the TLV and TLC. Both cross-sectional EDSS (EDSS_0_) and EDSS change after 2 years (ΔEDSS) were considered for this analysis.

Permutation tests (*N* = 5000) were used to statistically compare the magnitude of tract-specific correlations, each time by randomizing the order of the EDSS scores across patients. The obtained *p*-values were corrected for multiple comparisons across white matter tracts by using the Benjamini–Hochberg method.

First, to test which imaging biomarker obtained the highest clinical correlation, we compared correlation amplitudes from the tract-specific average absolute *z*-score (${\mu }_{|z|}$) (“magnitude of deviation from the norm") with correlations of the volume of absolute *z*-scores exceeding a threshold of two (${V}_{\left|z\right|>2}$) (“spatial extent of deviation from the norm”), hence defining the following null hypotheses:

$${H}_{0}:|c\left({\mu }_{\left|z\right|}^{\mathrm{NAWM}}, {\mathrm{EDSS}}_{0}\right)|-|c\left({V}_{\left|z\right|>2}^{\mathrm{NAWM}}, {\mathrm{EDSS}}_{0}\right)|=0,$$

$${H}_{0}:|c\left({\mu }_{\left|z\right|}^{\mathrm{lesions}}, {\mathrm{EDSS}}_{0}\right)|-|c\left({V}_{\left|z\right|>2}^{\mathrm{lesions}}, {\mathrm{EDSS}}_{0}\right)|=0,$$

where *c*(*X*, EDSS_0_) represents the correlation between the imaging biomarker *X* and the cross-sectional EDSS.

Second, we assumed that due to their ability to reflect the extent and severity of microstructural tissue damage, imaging biomarkers reflecting T1 abnormalities yield stronger correlations with EDSS, compared to tract-specific lesion load. Hence, we compared correlation amplitudes obtained from tract-specific z-score metrics to lesion load per tract with cross-sectional EDSS using one-sided permutation tests under these null hypotheses:

$${H}_{0}:|c\left({\mu }_{\left|z\right|}^{\mathrm{NAWM}}, {\mathrm{EDSS}}_{0}\right)|-|c\left(\mathrm{LV},\mathrm{ EDS}{\mathrm{S}}_{0}\right)|\le 0,$$

$${H}_{0}:|c\left({\mu }_{\left|z\right|}^{\mathrm{lesions}}, {\mathrm{EDSS}}_{0}\right)|-|c\left(\mathrm{LV}, {\mathrm{EDSS}}_{0}\right)|\le 0.$$

Third, to test whether the imaging biomarkers better reflected current or future EDSS scores (progression), correlations between tract-specific metrics and cross-sectional EDSS were also compared to EDSS change (ΔEDSS) using two-sided permutation tests, with the following null hypotheses:

$${H}_{0}:|c\left({\mu }_{\left|z\right|}^{\mathrm{NAWM}}, {\mathrm{EDSS}}_{0}\right)|-|c\left({\mu }_{\left|z\right|}^{\mathrm{NAWM}}, \Delta \mathrm{EDSS}\right)|=0,$$

$${H}_{0}:|c\left({\mu }_{\left|z\right|}^{\mathrm{lesions}}, {\mathrm{EDSS}}_{0}\right)|-|c\left({\mu }_{\left|z\right|}^{\mathrm{lesions}}, \Delta \mathrm{EDSS}\right)|=0,$$

$${H}_{0}:|c\left(\mathrm{LV},\mathrm{ EDS}{\mathrm{S}}_{0}\right)|-|c\left(\mathrm{LV}, \Delta \mathrm{EDSS}\right)|=0.$$

## Results

When measured in the same patient, T1 values derived from GRAPPAx3 and CSx4 MP2RAGE sequences yielded comparable results, in accordance with the previous reports [23] (see Supplementary Fig. 1).

Overall, seven patients with progressive MS were discarded from the analysis due to insufficient registration quality, caused by the presence of severe atrophy. Supplementary Fig. 2 provides examples of good and bad registration quality.

**Fig. 2:**
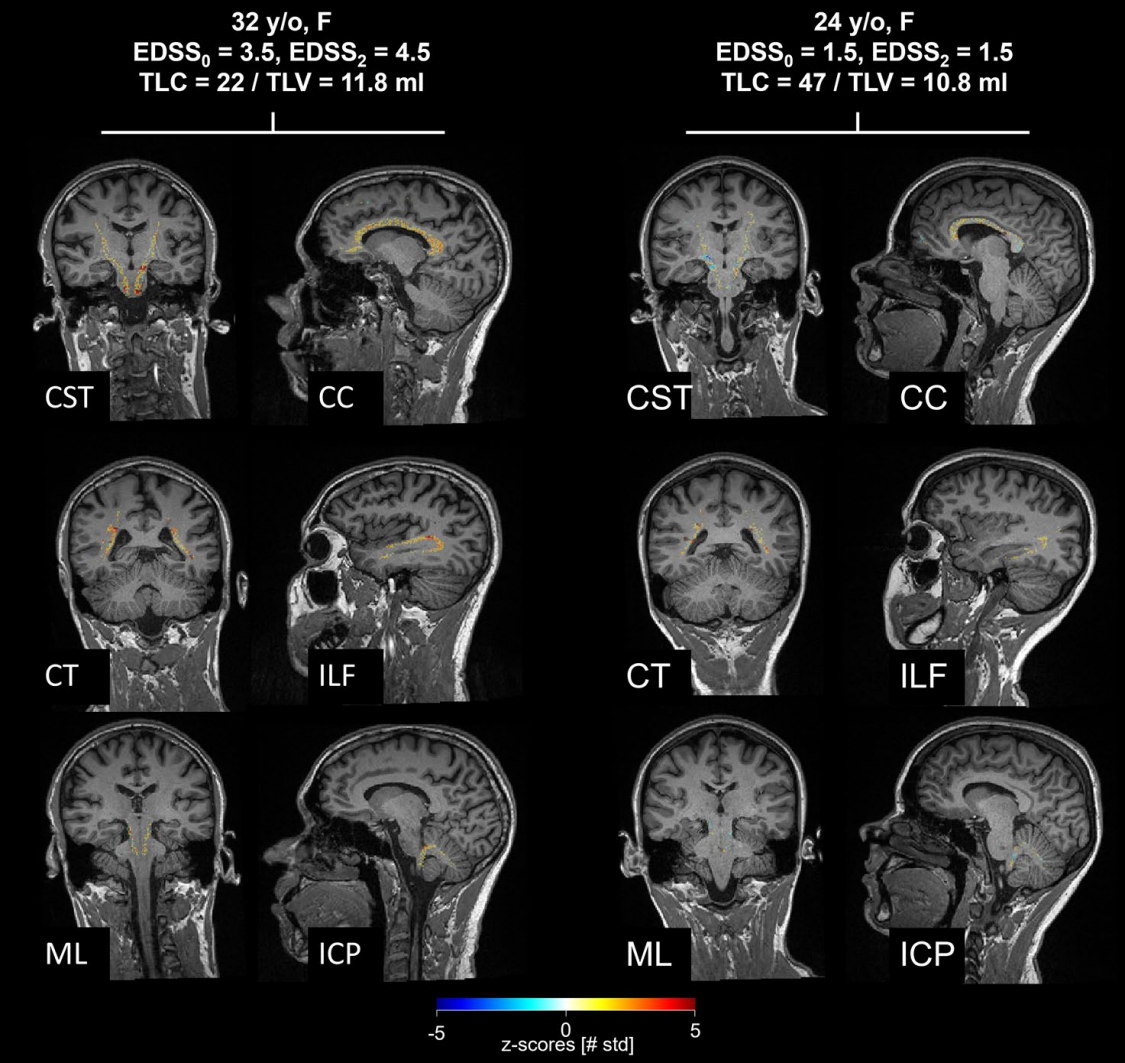
White matter tract-specific T1 *z*-scores shown in two example early MS patients. Despite similar total lesion load, the patient on the left, whose EDSS increased over 2 years, presented more severe alterations of the NAWM compared the patient on the right, who was clinically stable. *CST* cortico-spinal tract; *CT* corticostriatal pathway; *ML* medial lemniscus; *CC* corpus callosum; *ILF* inferior longitudinal fasciculus; *ICP* inferior cerebellar peduncle; *SST* spinothalamic tract; *TLC* total lesion count; *TLV* total lesion volume; *EDSS* expanded disability status scale

T1 *z*-score maps estimated in WM tracts are shown in Fig. 2 for two representative patients with early MS in six example WM tracts. The two patients are characterized by similar TLV (11.8 and 10.8 mL), but different EDSS evolution: while the first patient’s EDSS increased by 1 over 2 years, the second patient remained stable over the same period. When looking at the *z*-score maps, the first patient was characterized by larger T1 deviations in most WM tracts compared to the second patient, particularly in NAWM.

### General observations

Tract-specific average absolute *z*-scores (${\mu }_{|z|}$) and volumes of *z*-scores exceeding a threshold of 2 (${V}_{\left|z\right|>2}$) resulted in comparable correlations with EDSS when estimated both in lesions and NAWM. The difference in correlation when estimating ${\mu }_{|z|}$ compared to ${V}_{\left|z\right|>2}$ did not result in significant differences after correction for multiple comparisons. Hence, we only report the results of correlation comparisons in terms of ${\mu }_{|z|}$.

**Fig. 3:**
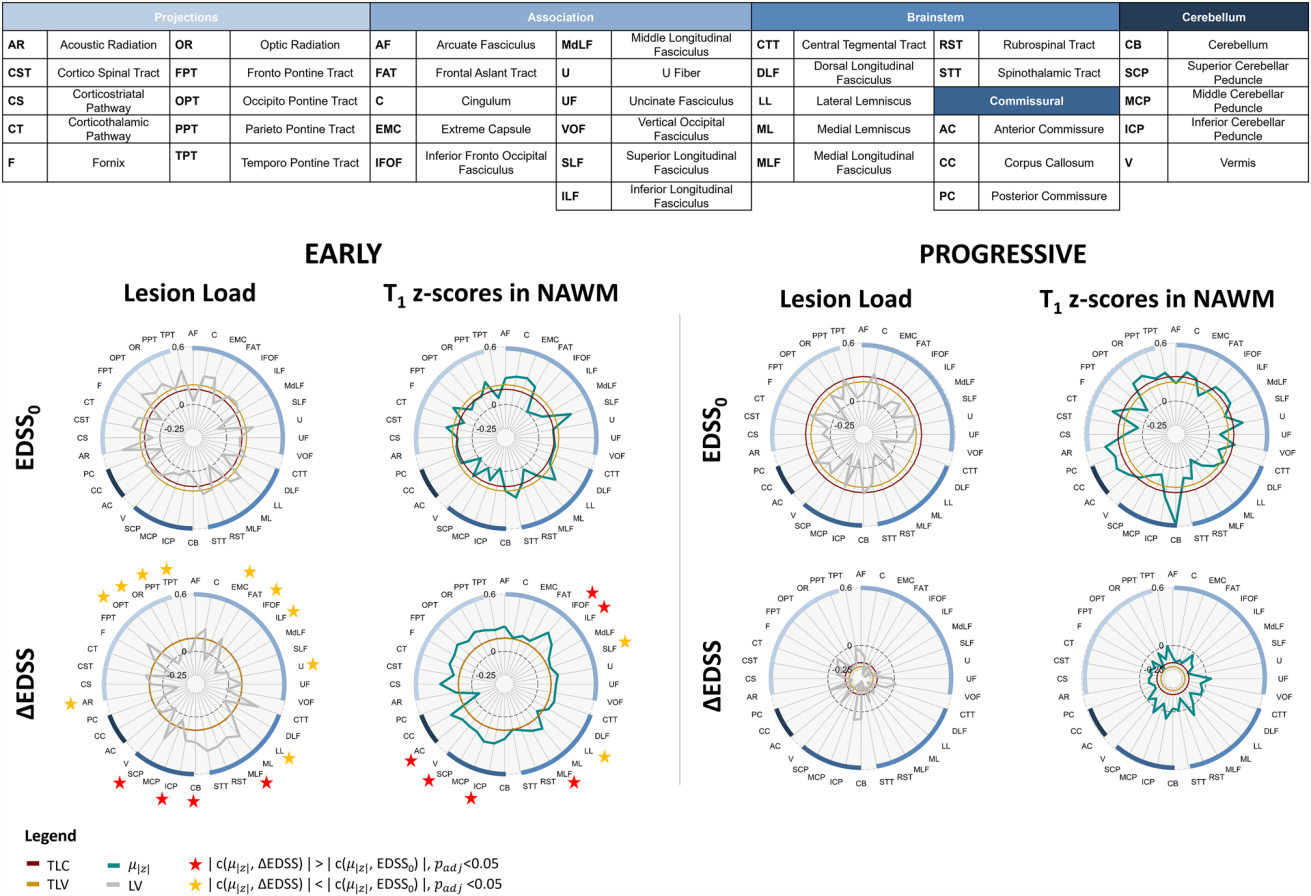
Spearman correlations between white matter tract-specific metrics and EDSS in early (left half) and progressive MS (right half) cohorts. We estimated correlation of tract-specific lesion volume (LV, gray, first column) and T1 abnormalities in the NAWM (second column). We computed the average absolute *z*-score value in each tract (${\mu }_{|z|}$, teal). Correlations to EDSS_0_ are shown in top rows and ΔEDSS in bottom rows. The inner circles represent the correlation of TLC (red) and TLV (gold). Red (/yellow) stars highlight tracts that yield to significantly higher (/lower) correlations to ΔEDSS compared to cross-sectional EDSS. Tract abbreviations are provided in the table. *NAWM* normal-appearing white matter; *EDSS*_*0*_ expanded disability status scale, *ΔEDSS* change in EDSS after 2 years; *TLC* total lesion count; *TLV* total lesion volume; *LV* tract-specific lesion volume

Figure 3 shows the Spearman correlations computed between tract-specific metrics (lesion load and T1 *z*-scores in NAWM) and both EDSS measures (EDSS_0_ and ΔEDSS) in both cohorts, together with abbreviations for all tracts mentioned hereafter. The correlations obtained from metrics reflecting T1 abnormalities specifically in lesioned tissue are reported in Supplementary Fig. 3. The posterior commissure (PC) and dorsal longitudinal fasciculus (DLF) were removed from statistical analyses when considering lesion-related metrics due to an insufficient number of patients presenting lesions in these tracts.

In early MS, estimating tract-specific lesion load yielded to higher correlations with EDSS_0_ compared to TLV (ρ = 0.20, *p* = 0.16) and TLC (ρ = 0.16, *p* = 0.28) for 17/37 WM tracts, whereas it did not yield stronger correlations in the progressive cohort TLV (ρ = 0.20, *p* = 0.19), TLC (ρ = 0.25, *p* = 0.09) for most tracts (35/37).

The estimation of T1 *z*-scores in NAWM significantly improved the correlation with cross-sectional EDSS compared to tract-specific lesion load for two tracts in the early cohort: the frontal aslant tract (FAT) and the superior longitudinal fasciculus (SLF); and for five tracts in the progressive cohort: the middle longitudinal fasciculus (MdLF), the inferior cerebellar peduncle (ICP), the optic radiation (OR), the temporo-pontine tract (TPT), and the rubrospinal tract (RST), as reported in Supplementary Tables 2 and 3. The estimation of T1 *z*-scores inside lesions yielded a substantial, yet non-significant, increase in correlation to EDSS_0_ compared to tract-specific lesion load for some supratentorial tracts in the progressive cohort, whereas no notable difference could be observed for the early cohort.

### Early MS cohort

Table 3 reports the significant results obtained from the correlation comparisons between ΔEDSS and EDSS_0_ in early MS patients. Overall, a drop in correlations was observed for supratentorial tract-specific lesion load when considering ΔEDSS compared to EDSS_0_. This difference was significant for nine association or projection pathways (highlighted by yellow stars in Fig. 3): the acoustic radiation (AR), the occipito-pontine tract (OPT), the optic radiation (OR), the parieto-pontine tract (PPT), the temporo-pontine tract (TPT), the extreme capsule (EMC), the inferior fronto-occipital fasciculus (IFOF), the inferior longitudinal fasciculus (ILF) and the U fibers (U), as well for one brain stem tract: the lateral lemniscus (LL). On the other hand, higher correlations were observed for four infratentorial tracts (highlighted with red stars in Fig. 3): the superior cerebellar peduncle (SCP), the cerebellum (CB), the inferior cerebellar peduncle (ICP) and the medial longitudinal fasciculus (MLF). The five tracts that yielded to the highest correlations to EDSS_0_ were mostly projection pathways [AR (ρ = 0.39, *p* = 0.006), TPT (ρ = 0.37, *p* = 0.011), EMC (ρ = 0.31, *p* = 0.03), FPT (ρ = 0.35, *p* = 0.015), C (ρ = 0.30, *p* = 0.04)], whilst those with the strongest correlations to ΔEDSS were infratentorial tracts [ML (ρ = 0.37, *p* = 0.01), STT (ρ = 0.37, *p* = 0.011), CTT (ρ = 0.35, *p* = 0.02), RST (ρ = 0.35, *p* = 0.02), SCP (ρ = 0.32, *p* = 0.03)].

**Table 3:** Significant results obtained from correlation comparisons between EDSS_0_ and ΔEDSS in the early MS cohort

	$c\left(\mathrm{LV},\mathrm{ EDS}{\mathrm{S}}_{0}\right)$	$c\left(\mathrm{LV}, \Delta \mathrm{EDSS}\right)$	${p}_{\mathrm{adj}}$		$c\left( {\upmu }_{\left|\mathrm{z}\right|}^{\mathrm{NAWM}},{\mathrm{EDSS}}_{0}\right)$	$c\left({\upmu }_{\left|\mathrm{z}\right|}^{\mathrm{NAWM}},\Delta \mathrm{EDSS},\right)$	${p}_{\mathrm{adj}}$
MLF	−0.02	**0.30**	0.003	IFOF	0.006	**0.36**	0.018
CB	0.07	**0.26**	0.003	ILF	0.04	**0.29**	0.039
ICP	0.01	**0.26**	0.004	MLF	0.03	**0.22**	0.039
SCP	0.07	**0.32**	0.046	ICP	−0.02	**0.28**	0.039
AR	**0.39**	−0.15	0.015	AC	−0.01	**0.23**	0.041
OPT	**0.26**	−0.06	0.018	SCP	0.01	**0.23**	0.047
OR	**0.30**	−0.02	0.003	SLF	**0.37**	0.16	0.039
TPT	**0.37**	−0.01	0.003	LL	**0.32**	0.1	0.036
PPT	**0.19**	0.04	0.035				
EMC	**0.31**	−0.08	0.006				
IFOF	**0.21**	0.03	0.027				
ILF	**0.23**	−0.05	0.027				
U	**0.28**	0.008	0.003				
LL	**0.29**	0.14	0.014				

For each tract, the highest correlation is highlighted in bold

In contrast, a substantial increase was observed for most tracts when correlating NAWM z-scores metrics with ΔEDSS compared to cross-sectional EDSS_0_. However, after correction for multiple comparisons, only six tracts exhibited a significant increase: the IFOF, the ILF, the MLF, the ICP, the SCP and the anterior commissure (AC). On the other hand, the lateral lemniscus (LL) and the superior longitudinal fasciculus (SLF) presented a significantly stronger correlation with EDSS_0_ compared to ΔEDSS. In contrast to tract-specific lesion load, the lowest correlations between ${\upmu }_{\left|\mathrm{z}\right|}^{\mathrm{NAWM}}$ and ΔEDSS were found in the brainstem and commissural tracts (PC (ρ = −0.06), LL (ρ = 0.09), STT (ρ = 0.14), C (ρ = 0.15), SLF (ρ = 0.16), all with *p* > 0.05), whilst cerebellar and projection pathways yielded comparatively high correlations.

When considering T1 *z*-score metrics estimated in lesions (reported in Supplementary Fig. 3 and Supplementary Table 2), the correlations were significantly higher for ∆EDSS compared to EDSS_0_ for the MLF, the SCP and the ICP. The strongest correlations to ∆EDSS were again observed for infratentorial tracts [SCP (ρ = 0.40, *p* = 0.0058), ML (ρ = 0.36, *p* = 0.013), RST (ρ = 0.35, *p* = 0.01), STT (ρ = 0.35, *p* = 0.01), CTT (ρ = 0.35, *p* = 0.02)].

### Progressive MS cohort

In the progressive cohort, the lesion load in tracts did not result in significantly different correlations when associated to ∆EDSS compared to EDSS_0_. Compared to the early MS cohort, the correlations to EDSS_0_ were smaller (ρ < 0.3) and none was statistically significant. Additionally, correlation values differed greatly between white matter tracts belonging to the same pathways and no specific spatial pattern could be identified.

When considering ∆EDSS, a substantial drop in correlation was observed for all tracts both in terms of tract-specific lesion load and NAWM *z*-scores compared to EDSS_0_. This resulted in extremely poor and often negative correlations and often negative correlations.

Finally, when considering *z*-scores metrics extracted in lesions (reported in Supplementary Fig. 3 and Supplementary Table 2), the correlations were overall decreased for ∆EDSS compared to EDSS_0_. However, these differences were never significant. Again, similar to what was observed for tract-specific lesion load, the infratentorial tracts, and particularly brainstem tracts, were among the most weakly correlated both with EDSS_0_ (SCP = −0.01, FAT = 0.04, ICP = −0.05, RST = −0.05, MCP = 0.07, all with *p* > 0.05) and ∆EDSS (*U* = 0.002, ICP = 0.006, CB = −0.02, LL = −0.02, FPT = −0.02, all with *p* > 0.05).

## Discussion

We proposed a technique to estimate microstructural properties of brain tissue along WM tracts which were extracted from a connectivity atlas, i.e., without the need of additional diffusion imaging. A normative T1 atlas was used to extract patient-specific voxel-wise deviations while accounting for sex and age, as both are known to impact T1 values in the brain [29, 33].

Overall, our results suggest that the spatial distribution of lesions and microstructural alterations in WM tracts can improve the correlation with both cross-sectional disability and its evolution over 2 years compared to total lesion volume and count. In contrast to the early MS patients, the differences in correlation between tract-specific metrics and EDSS_0_ and ΔEDSS, respectively, were never significant in the progressive cohort. This distinction observed between the two disease stages can probably be explained by a higher disease activity in the earlier stages of the disease, whilst a more stationary phase is reached with longer disease durations. Additionally, the EDSS is an ordinal scale that is known to reflect disability in a non-linear way: depending on the disease stage, the same increase in EDSS is not reflected by the same severity of disability evolution. Hence, considering ΔEDSS as a measure of disease evolution bears limitations in terms of interpretability and comparability between MS groups. Furthermore, different therapeutic strategies might influence differently disability evolution: the use of more aggressive therapies in the progressive MS cohort is likely to have an impact on ΔEDSS and might, therefore, contribute to explaining the lower correlations.

Of particular interest is that in patients with early-stage MS, the contribution to the characterization of current and future disability appears to be very different depending on the WM pathway that is considered as different patterns were observed for association and projection pathways compared to infratentorial tracts (brainstem and cerebellar bundles).

The presence of lesions in infratentorial tracts correlated more strongly with disability change than cross-sectional EDSS, while an opposite trend was observed for many projection and association pathways. These findings suggest that, when estimated in supratentorial pathways, tract-specific lesion load is mainly contributing to explaining the current disability of the patient, whilst the presence of lesions in infratentorial tracts has a larger impact on the future evolution of the disease course. These results are in concordance with previous work showing the predictive power of infratentorial lesions to longitudinal disability [34]. One possible explanation is Wallerian degeneration [17], that has been widely studied in the context of MS [19, 20, 35]. Such a retrograde alteration of axonal pathways might have a bigger impact on brain function when infratentorial tracts are affected by lesions, since damage occurring at a more proximal level of the axonal pathway is more likely to involve a larger portion of fibers than damage in distal sections. Another hypothesis is that brain plasticity could be more effectively compensating alterations of high-level brain functions, typically modulated by association and projection pathways (e.g., motor, and sensory systems), than autonomic functions that are regulated in infratentorial regions (e.g., urination, awareness).

The evaluation of T1 alterations in tract-specific NAWM resulted in stronger correlations with disability change for some supratentorial and cerebellar pathways, compared to cross-sectional EDSS, except for brainstem tracts. These findings suggest that the subtle tissue alterations found in the NAWM are more predictive of future disease activity than to lesion load. Similar observations were made evaluating periventricular gradients of T1 alterations in the NAWM compared to regional lesion volume [9].

When comparing the correlations obtained from tract-specific z-score metrics in lesions, despite being comparatively high, the differences were rarely significant. One probable explanation is that only few patients exhibit lesions in certain tracts, therefore driving the correlation (see Supplementary Fig. 4). However, when performing permutations, these correlations do not hold and the correlation difference drops.

The limitations of the proposed technique include the need for a non-linear registration between the native patient space and the SST that could result in false-positive z-scores notably at the boundaries of the white matter, as also reported originally by Piredda et al. [29]. This is of particular concern when considering patients with severe atrophy, hence limiting the applicability of the developed method in progressive patients. Further, the goodness of fit of the regression model used to estimate normative T1 values depends on the size and the age span of the healthy cohort. Therefore, estimating z-score values in patients whose age is outside the range spanned by the healthy cohort could result in loss of precision. By using a tractography atlas to model structural connectivity, our method does not account for individual anatomical differences in white matter tracts. Such differences can result from both normal aging process and adaptative mechanisms that occur in the presence of pathology, such as Wallerian degeneration.

By considering the absolute values of *z*-score maps, the same weight was given to positive and negative deviations in T1 values. While the vast majority of significant T1 *z*-scores were positive, sparsely distributed negative *z*-scores were also sporadically observed. Future work will focus in investigating the presence and possible cause of such T1 decrease, also in combination with other relaxometry atlases. Further, the spatial distribution of T1 abnormalities along white matter tracts could also be investigated as a new measure of connectivity damage.

In conclusion, estimating T1 abnormalities both in lesions and NAWM along white matter tracts improves the correlation with current and future disability in MS in patients at an early disease stage. Furthermore, our approach allows to estimate microstructural changes in strategic brain locations in a clinical setting without requiring lengthy advanced diffusion imaging, hence within clinically compatible acquisition and processing times. Our results suggest that, while the presence of infratentorial lesions correlates strongly with cross-sectional disability, the evaluation of T1 alterations in the NAWM is more predictive of future disability change in 2 years in early MS patients. Hence, our method provides promising additional prognostic information to the presence of conventionally accepted negative prognostic markers (e.g., infratentorial and intramedullary), that could contribute to identifying early MS patients with higher risk of progression in a context of therapy planning.

### Supplementary Information

Below is the link to the electronic supplementary material.

Supplementary file1 (DOCX 3501 KB)

## Data Availability

Anonymised data not published in this article will be made available upon reasonable request from a qualified investigator.
