# Supplementary material for: Tract-wise microstructural analysis informs on current and future disability in early multiple sclerosis
Source: J Neurol. 2023 Oct 11;271(2):631–41. doi: 10.1007/s00415-023-12023-3 (PMC10827809; doi:10.1007/s00415-023-12023-3)

**Supplementary materials :**

**Supplementary Figure 1:** T1 maps (top) and z-score maps (bottom) derived from the MP2RAGE sequence in the same multiple sclerosis patient using different acceleration techniques. The median and interquartile range of the z-score difference in the white matter between the two maps was 0.08 [0.90]. Left: 4-fold compressed sensing (CS) acceleration. Middle: 3-fold GRAPPA acceleration. Right: voxel-wise difference map between acceleration techniques.


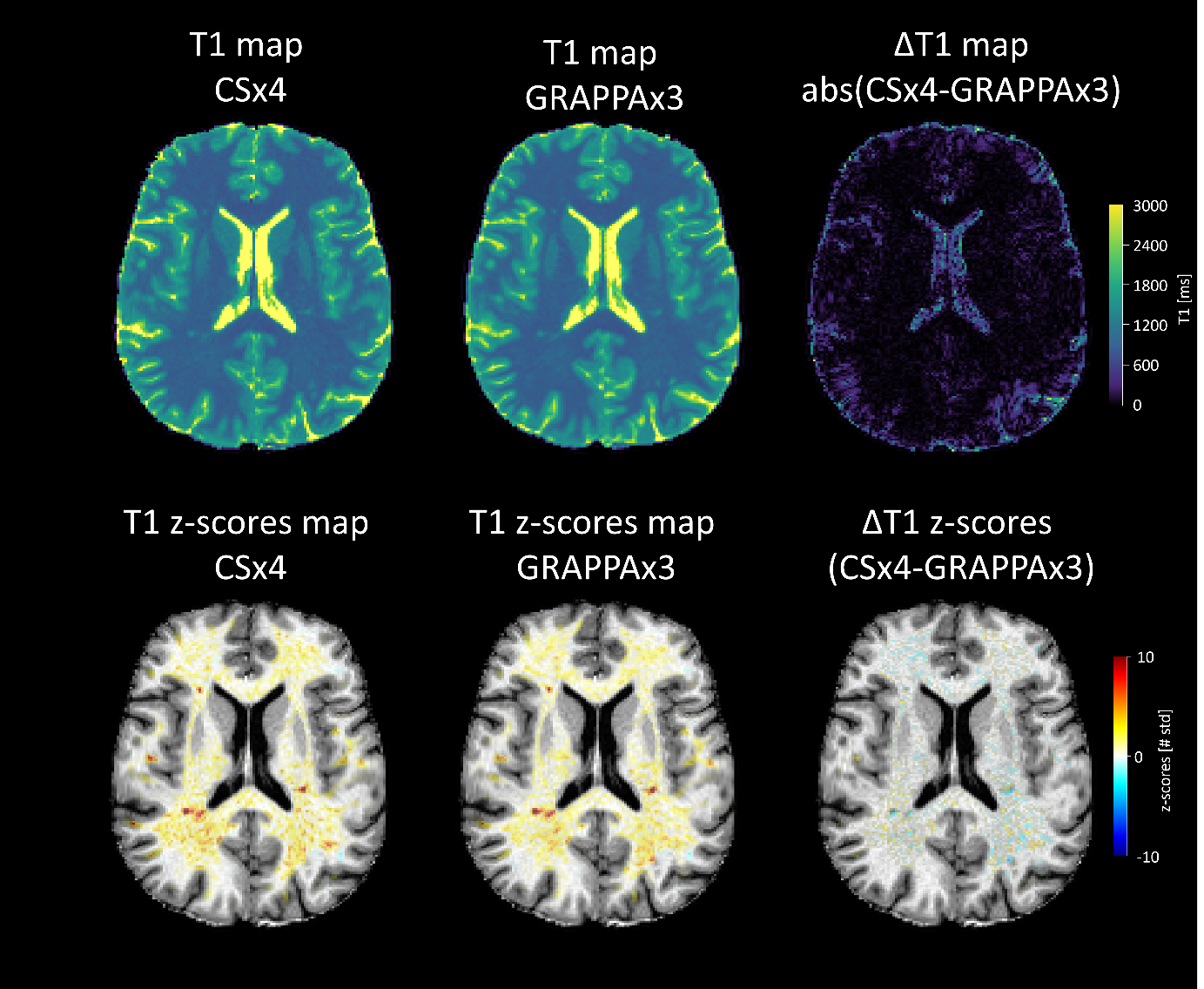


**Supplementary Figure 2:** Examples of good (top) and bad registration caused by severe brain atrophy (bottom) between native MP2RAGE space and SST in two progressive patients. Left: the UNI anatomical contrast from the MP2RAGE sequence registered onto the study specific template. Middle: SST. Right: The corpus callosum tract mask overlayed onto the SST for both patients.The patient with the bad registration quality was discarded from the analysis based on our inclusion criterion.

***
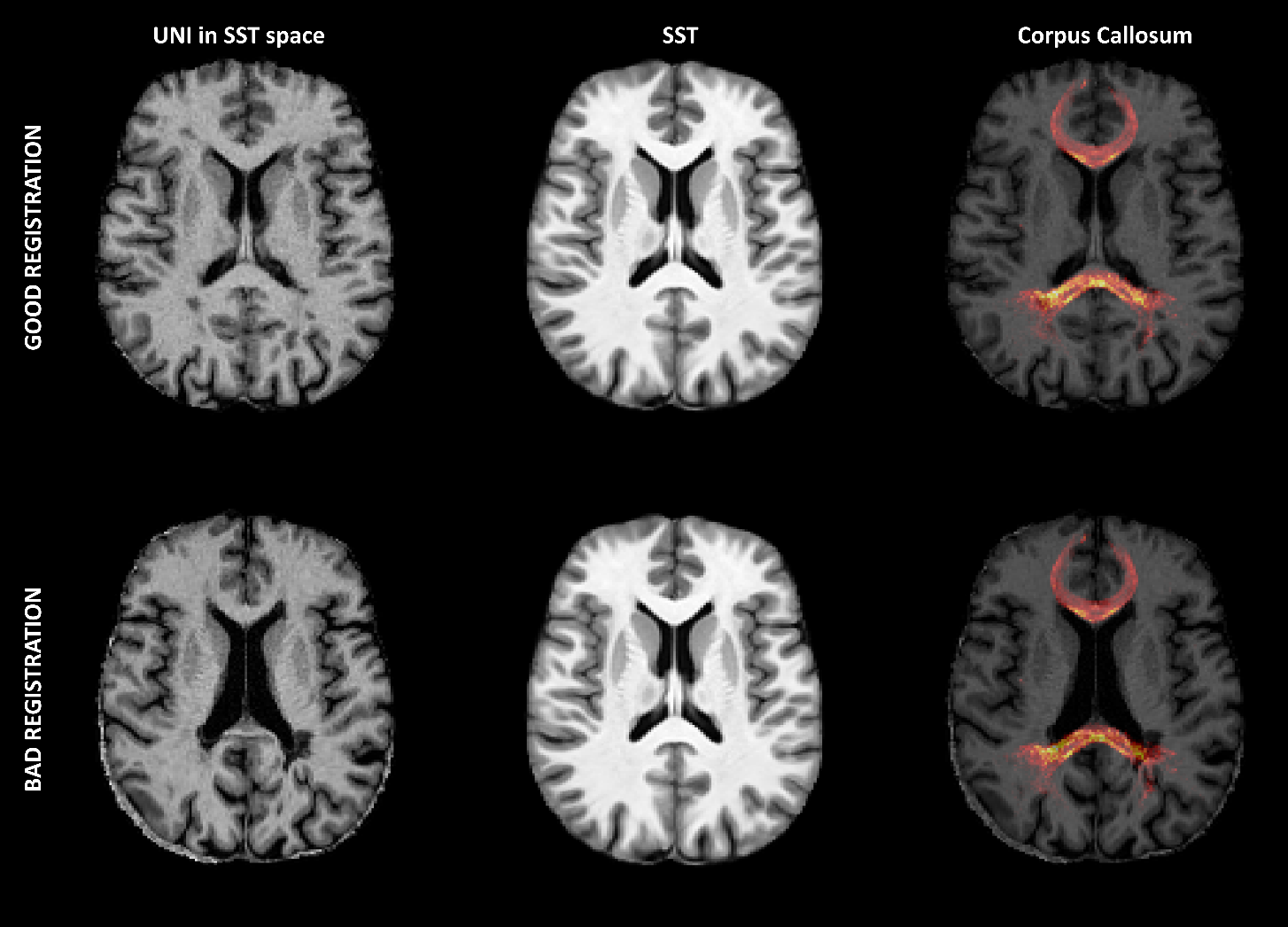
***

**Supplementary Figure 3:** Spearman correlations between white-matter tract-specific metrics and EDSS in early (left half) and progressive MS (right half) cohorts. We estimated correlation of tract-specific T1 abnormalities in the lesions. We computed the average absolute z-score value in each tract (orange) and the volume of the tracts with an absolute z-score greater than 2 (teal). Correlations to EDSS_0_ are shown in top rows and ΔEDSS in bottom rows. The inner circles represent the correlation of TLC (red) and TLV (gold).

Abbreviations: Tract abbreviations are provided in the table. NAWM: normal appearing white matter, EDSS_0_: expanded disability status scale, ΔEDSS: change in EDSS after two years, TLC: total lesion count, TLV: total lesion volume.


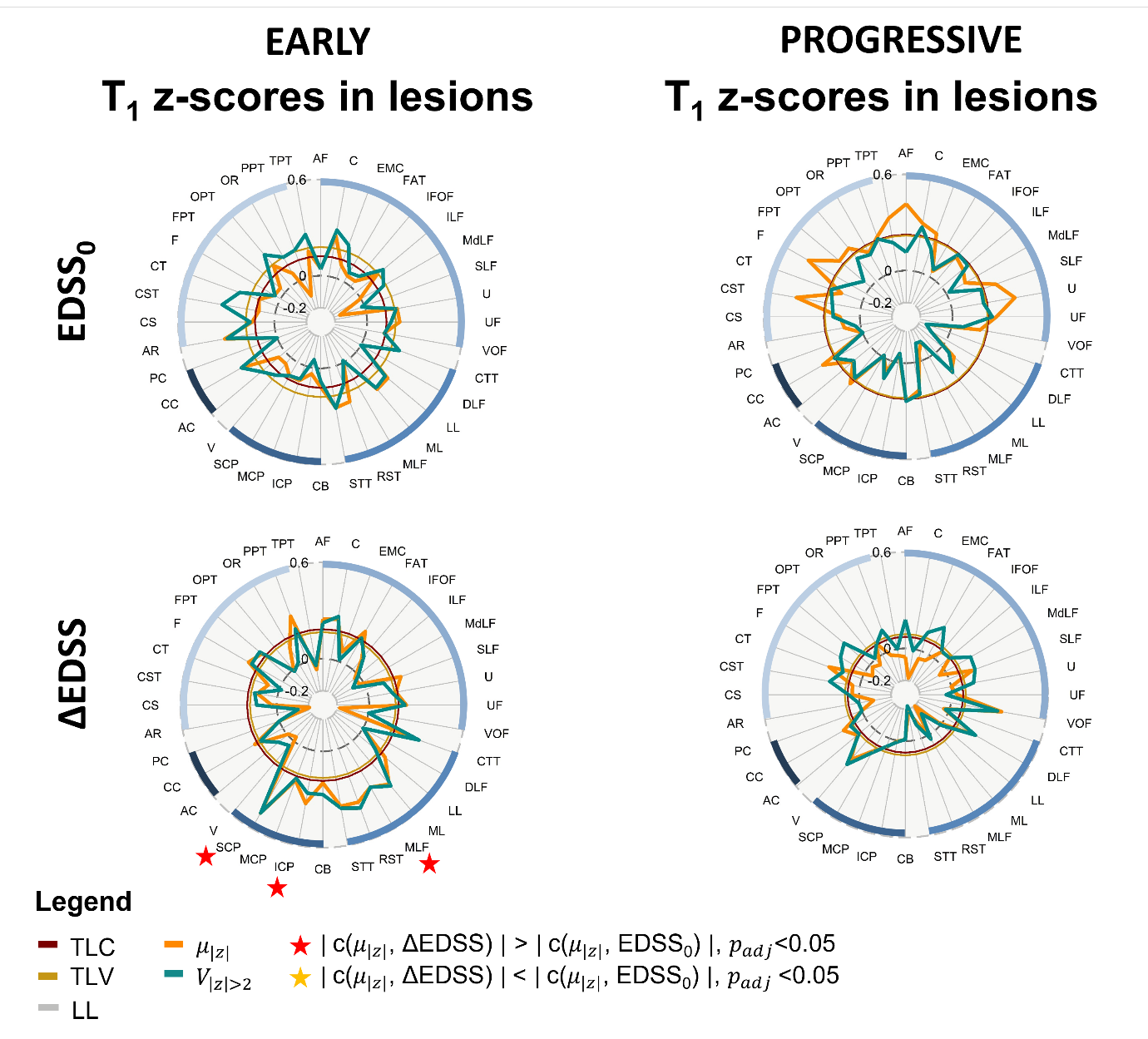


**Supplementary Table 1.** Treatment status at both time points for early and progressive MS cohort. First line treatments included interferon beta, dimethyl fumarate, teriflunomide, glatiramer acetate and ponesimod. Second line treatments included ocrelizumab, fingolimod, natalizumab, rituximab, sipondimod, ofatumumab, cladribine and alemtuzumab. NT: no treatment.

|  | $\mathbf{Baseline}$ | Follow-up | $\mathbf{N}$ |
| --- | --- | --- | --- |
| *Early MS* | First line | First line | 30 |
|  | First line | Second line | 10 |
|  | NT | First line | 2 |
|  | First line | NT | 3 |
|  | NT | Second line | 1 |
|  | NT | NT | 1 |
| *Progressive MS* | First line | First line | 8 |
|  | First line | Second line | 1 |
|  | Second line | Second line | 29 |
|  | NT | NT | 10 |
|  | NT | Second line | 2 |
|  | Second line | NT | 1 |

**Supplementary Table 2:** Significant results obtained from correlation comparisons for the early MS cohort, when comparing T1 z-scores metrics to tract-specific lesion volume (LL) (top), or estimating T1 z-scores in lesions (bottom).

|  | $\mathbf{c}\left( \mathbf{EDS}\mathbf{S}_{\mathbf{0}}\mathbf{,}\boldsymbol{\mu}_{\left\vert\mathbf{z} \right\vert}^{\mathbf{NAWM}} \right)$ | $\mathbf{c}\left( \mathbf{EDS}\mathbf{S}_{\mathbf{0}}\mathbf{,}\boldsymbol{LL} \right)$ | $\mathbf{p}_{\mathbf{adj}}$ |
| --- | --- | --- | --- |
| *FAT* | **0.27** | 0.07 | 0.013 |
| *SLF* | **0.37** | -0.07 | 0.006 |
|  | $\mathbf{c}\left( \mathbf{EDS}\mathbf{S}_{\mathbf{0}}\mathbf{,}\boldsymbol{\mu}_{\left\vert\mathbf{z} \right\vert}^{\mathbf{lesions}} \right)$ | $\mathbf{c}\left( \boldsymbol{\Delta EDSS,}\boldsymbol{\mu}_{\left\vert\mathbf{z} \right\vert}^{\mathbf{lesions}} \right)$ | $\mathbf{p}_{\mathbf{adj}}$ |
| *MLF* | -0.01 | **0.30** | 0.001 |
| *SCP* | 0.10 | **0.49** | 0.001 |
| *ICP* | 0.04 | **0.33** | 0.001 |

**Supplementary Table 3.** Significant results obtained from correlation comparisons for the progressive MS cohort, when comparing T1 z-scores metrics to tract-specific lesion volume (LL)

|  | $\mathbf{c}\left( \mathbf{EDS}\mathbf{S}_{\mathbf{0}}\mathbf{,}\boldsymbol{\mu}_{\left\vert\mathbf{z} \right\vert}^{\mathbf{NAWM}} \right)$ | $\mathbf{c}\left( \mathbf{EDS}\mathbf{S}_{\mathbf{0}}\mathbf{,}\boldsymbol{LL} \right)$ | $\mathbf{p}_{\mathbf{adj}}$ |
| --- | --- | --- | --- |
| *MdLF* | **0.30** | 0.00 | 0.024 |
| *ICP* | **0.31** | -0.08 | 0.004 |
| *TPT* | **0.30** | 0.04 | 0.004 |
| *RST* | **0.14** | 0.05 | 0.026 |
| *OR* | **0.35** | 0.07 | 0.023 |

**Supplementary Figure 4:** Correlation plot for two example tracts and both EDSS_0_ (left) and ΔEDSS (right) for the early MS cohort. Top. Optic radiation (OR) Bottom: Medial lemniscus (ML)


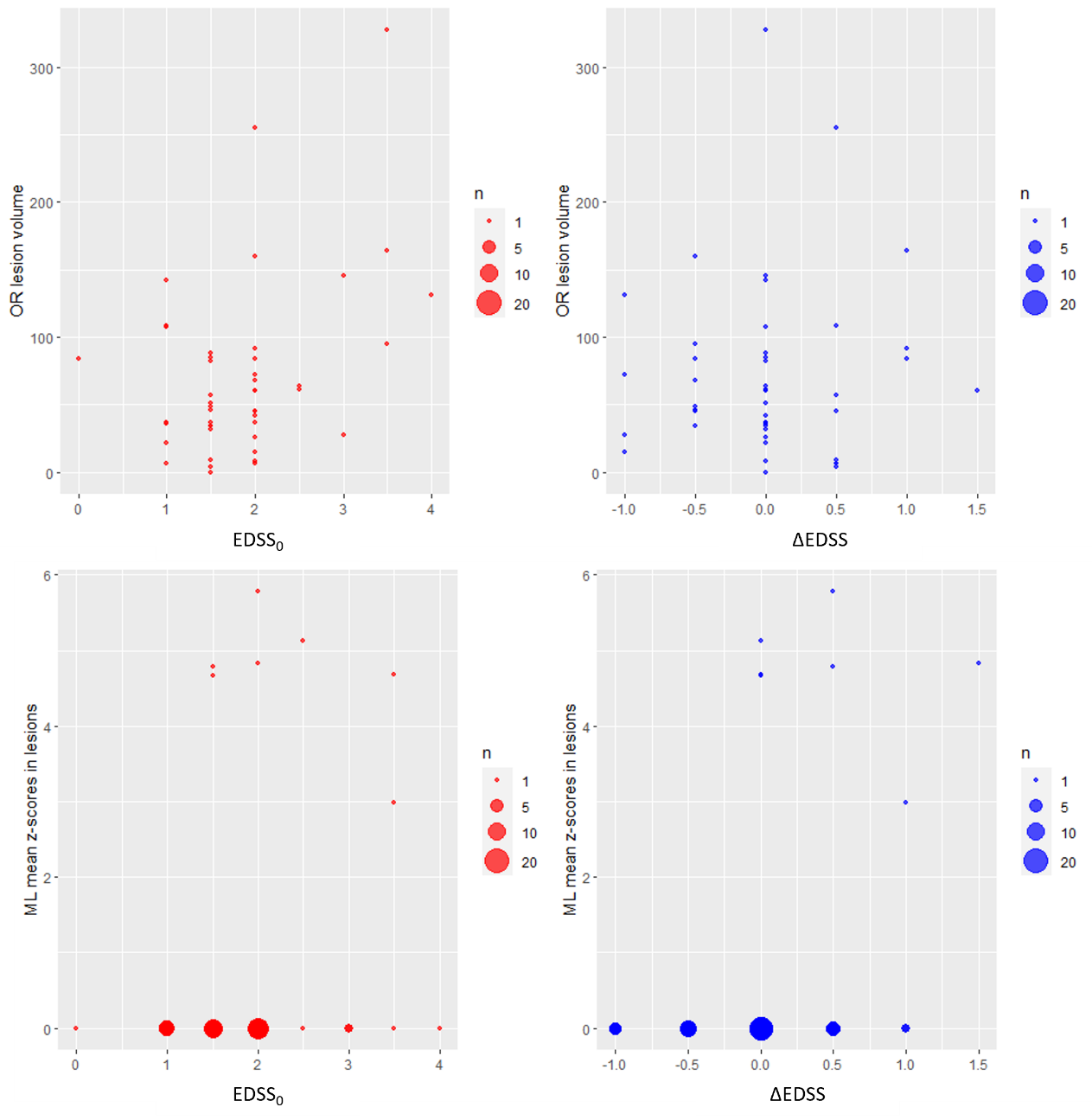

Supplement: Supplementary file 1 — Supplementary file1 (DOCX 3501 KB) [file 415_2023_12023_MOESM1_ESM.docx]
